# Frequency and Predictors of HIV-1 Co-receptor Switch in Treatment Naive Patients

**DOI:** 10.1371/journal.pone.0080259

**Published:** 2013-11-07

**Authors:** Virginie Mortier, Kenny Dauwe, Leen Vancoillie, Delfien Staelens, Filip Van Wanzeele, Dirk Vogelaers, Linos Vandekerckhove, Kristen Chalmet, Chris Verhofstede

**Affiliations:** 1 Aids Reference Laboratory, Department of Clinical Chemistry, Microbiology and Immunology, Ghent University, Gent, Belgium; 2 Aids Reference Center, Department of General Internal Medicine and Infectious Diseases, Ghent University Hospital, Gent, Belgium; Helmholtz Zentrum Muenchen - German Research Center for Environmental Health, Germany

## Abstract

**Background:**

Determination of HIV-1 co-receptor use is a necessity before initiation of a CCR5 antagonist but the longevity of a CCR5-use prediction remains unknown.

**Methods:**

Genotypic co-receptor tropism determination was performed in 225 newly diagnosed individuals consulting an AIDS Reference Centre. Samples were collected at diagnosis and at initiation of antiretroviral therapy or just before closure of the study for patients who did not initiate therapy. For individuals with a discordant tropism prediction on the two longitudinal samples, analysis of intermediate samples and single genome sequencing of proviral DNA was performed to confirm the tropism switch. Deep sequencing was done to identify minor CXCR4 or CCR5-using populations in the initial sample.

**Results:**

Overall, tropism switches were rare (7.6%). Only a geno2pheno false positive rate of <50% at baseline was retained as predictive for a subsequent switch from CCR5-use only to predicted CXCR4-use. Minor CXCR4-using virus populations were detected in the first sample of 9 of the 14 R5-to-X4 switchers but the subsequent outgrowth of these minor populations was documented in only 3.

**Conclusions:**

With the current guidelines for treatment initiation at CD4^+^ T cell counts of <500 cells/mm^3^, co-receptor switch between diagnosis and starting antiretroviral therapy is rare. Patients with R5 viruses and a geno2pheno FPR of <50% are more prone to subsequent co-receptor switch than patients with an FPR of >50% and will need repeat tropism testing if initiation of maraviroc is considered and previous testing dates from more than a year before.

## Introduction

The human immunodeficiency virus type 1 (HIV-1) is dependent on binding to the CD4 receptor and a co-receptor, either CCR5 or CXCR4, for entry into target cells. The development of entry inhibitors has increased interest in co-receptor affinity or tropism. The only entry inhibitor currently FDA/EMA approved is the CCR5 antagonist maraviroc. This drug can be initiated only after excluding the presence of virus able to use CXCR4. Phenotypic as well as genotypic assays have been developed for co-receptor tropism analysis and both can be used to screen for maraviroc sensitivity [1,2]. Depending on the geographical region phenotypic or genotypic methods are more widely used.

In recently infected individuals in general CCR5-using (R5) variants are found. Progression of the infection can lead to the occurrence of CXCR4-using (X4) strains [3,4]. In the absence of combination antiretroviral therapy (ART) and in treatment experienced patients with a history of therapy failure, co-receptor switch has been observed in approximately half of subtype B infected individuals [5-9]. Detection of X4 variants is associated with accelerated CD4 decline, increased plasma HIV-1 RNA levels and hence with a faster disease progression [8,10-14]. The mechanism behind co-receptor switch is still largely unknown and the question whether co-receptor switch is cause or consequence of the accelerated disease progression remains unanswered.

Data suggests that the risk for tropism switch over time in patients with suppressed viremia is extremely low [15,16], but adequate data on the risk for a co-receptor switch pre-ART in the current era with relatively early start of medication, are still limited [17]. As a result, the DHHS guidelines and the European guidelines on tropism testing in clinical management of HIV-1 infected patients are unable to provide guidance on the durability of an R5 result [1,2]. 

The study described aimed at determining the prevalence of co-receptor switch over time in ART-naive individuals and at determining potential viral or patient characteristics that predict co-receptor switch. The results showed that pre-ART co-receptor tropism switch is rare. Only the false positive rate (FPR) of the geno2pheno co-receptor tropism prediction tool could be retained as predictive for faster co-receptor switch.

## Methods

### Ethics statement

The study was approved by the Ethics Committee of the University Hospital Ghent, EC number 2010/057. All analyses were performed on rest fractions of stored samples after written informed consent from the patients. Patient selection as well as sample analysis was done anonymously, researchers participating in the project were unable to couple back samples to original patients. 

### Study subjects

From 798 patients, newly registered at the AIDS Reference Centre (ARC) of Ghent University Hospital (Belgium) between January 2001 and December 2009, 244 patients were retrospectively selected based on the criteria that the patient had to be newly diagnosed and that a blood sample, collected within 1 year of diagnosis, as well as a blood sample collected at the start of ART or by the end of the study period (August 2011) if no ART was initiated, had to be available. The minimal time required between the two samples was 3 months.

Information on HIV transmission route, sex, age, origin, CD4^+^ T-cell count, viral load, and protease (PR) and reverse transcriptase (RT) resistance were retrieved anonymously from the patients’ files. Identification of drug resistance in the protease and *reverse transcriptase* genes was based on the Stanford HIV Drug Resistance database v6.2.0 (http://hivdb.stanford.edu). HIV-1 subtyping was performed using PR and RT sequences and the Smartgene subtyping tool (IDNS). 

### Co-receptor tropism determinations

Viral RNA was extracted from EDTA plasma with the High Pure viral RNA kit (Roche Applied Science). V3 amplification and population sequencing was performed as described before [18]. Sequencing products were analyzed on the ABI-Prism 3130XL Genetic Analyzer (Applied Biosystems). Proofreading was done with Smartgene (IDNS) and subsequently V3 nucleotide sequences were submitted to geno2pheno_[co-receptor_]2.0 (http://co-receptor.bioinf.mpi-inf.mpg.de/index.php) [19]. For classification as CCR5-using (R5) or CXCR4-using (X4), a false-positive rate (FPR) cut-off of 10% was applied in accordance with reports describing the use of this method to predict maraviroc susceptibility [20] and comparisons with phenotypic assays [21]. All V3 sequences generated for this study through population or deep sequencing are available on request. 

### Single genome sequencing of viral DNA

DNA was extracted from buffy coat cells using the QIAamp Blood Kit (Qiagen). For each DNA sample a 5- to 200-fold dilution series was created and several PCR reactions were run to determine the dilution for which only one third of identical PCR reactions provided a positive PCR product. Primers and conditions used for V3 amplifications were the same as the ones used for V3 amplification from RNA [18]. All individual PCR products were sequenced and all sequences with no more than 1 ambiguous nucleotide position were used for co-receptor tropism prediction. 

### Deep sequencing of V3

RNA extraction was performed on 200 to 500 µl of plasma, using the High Pure Viral RNA kit (Roche). 10 µl of extracted RNA was used in the initial RT-PCR reaction that was executed as described previously [18]. Amplicons generated during the RT-PCR reaction were then used as template for the nested PCR with primers 5’-TCAACHCAAYTRCTGTTAAATGG-3’ and 5’-ATTTCTGGRTCYCCKCCTG-3’ extended with Roche MIDs 1 to 8, 10, 11 and 13. The final amplification product spanned positions 6990 to 7336 (HxB2 numbering) of the envelope gene. Amplicons were purified using the AMPure XP DNA purification kit (Agencourt) and quantified using the Qubit dsDNA HS Assay (Life Technologies).

Emulsion PCR was done with an input of 1.5 copies per bead with the LibAemPCR kit (Roche). Deep sequencing was done on the 454 GS Junior platform (Roche) according to the manufacturer’s instructions. After data processing with the Roche instrument software, reads that passed the filtering were submitted to geno2pheno_[coreceptor]_ for tropism prediction. Data to determine the individual variants was filtered on an average Phred score above 25 with Mothur software [22] and aligned using Clustal W (Bioedit). Reads were trimmed to the V3 region and identical reads were clustered. Reads and their number of occurrence were listed for each plasma sample. Only variants with a minimum of 5 reads and representing at least 0.2 % of the total number of reads that passed the filtering, were retained.

### Phylogenetic analysis

V3 nucleotide sequences were manually aligned using BioEdit [23]. The best fitting nucleotide-substitution model was selected according to the Akaike Information Criterium (AIC) using jModeltest 0.1.1 [24]. Maximum likelihood estimated distances according to the chosen model, were used to construct neighbor-joining phylogenetic trees in PAUP* v4.0b10 (http://paup.csit.fsu.edu/) [25]. Bootstrap analysis was performed using the above mentioned conditions on 1000 replicates. The tree was rooted using the consensus V3 sequence of subtype B as outgroup. The tree was visualized using iTOL [26,27].

### CCR5 genotyping

To determine the presence of a deleterious 32-base pair (bp) deletion in CCR5, a fragment flanking the deletion was amplified from the patients’ genomic DNA after extraction with QIAamp DNA Blood minikit (Qiagen). Primers and amplification conditions were depicted by de Roda Husman et al. [5]. The reverse primer was fluorescently labeled with FAM to allow to define the length of the amplified products on an ABI-Prism 3130XL Genetic Analyzer (Applied Biosystems): wild type (wt)/wt: 239 bp, wt/∆32: 239 bp + 207 bp, ∆32/∆32: 207 bp.

### Statistical analysis

Groups were compared using a χ^2^ test for categorical variables and the Mann-Whitney *U* nonparametric test for continuous variables. The level of significance was set at p ≤ 0.05. Kaplan-Meier plots were drawn to present the co-receptor use evolution with time for the different patient groups. All data were analyzed using SPSS 19.0 software (SPSS).

## Results

### Co-receptor tropism determination and prevalence of co-receptor switch

**Figure 1:**
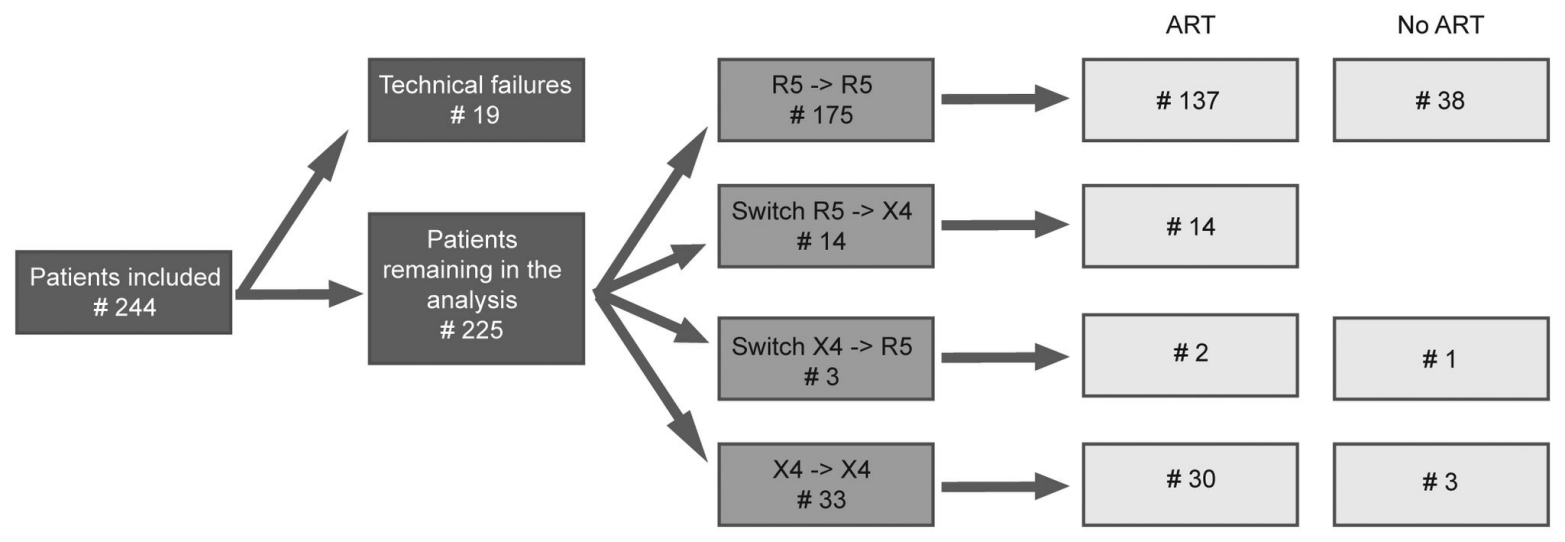
Overview of the number of patients included in each step of the analysis for each patient group: R5R5 and X4X4 non switchers, R5X4 and X4R5 switchers. #: number; ART: initiation of antiretroviral treatment (ART) during the study period; No ART: ART not initiated at the end of the study period.

Although the initial selection comprised 244 patients (Figure 1), 19 were omitted from the analysis because of failure to amplify the V3 loop or bad quality sequencing results. For the remaining 225 patients, the mean interval between the first and last sample analyzed was 32 months (IQR: 17-44); 30 months (IQR: 14-42) for the 183 patients who initiated ART and 42 months (IQR: 22-55) for the 42 patients who remained ART naive. 

Of the 225 patients included in the analysis, 189 (84%) were initially predicted as CCR5-using (R5) and 36 (16%) were predicted as CXCR4-using (X4). Analysis of the follow-up sample revealed that 175 (77.8%) remained CCR5-using (R5R5 = R5-using in first and second sample) and 33 (14.7%) remained CXCR4-using (X4X4) (Figure 1). R5X4 or X4R5 predictions were obtained for 17 individuals (7.6%) (Figure 1). For all 17 patients the tropism switch was confirmed after repeat analysis in triplicate and additional testing of at least 2 intermediate samples. A switch from CCR5- to CXCR4-use (R5X4) was seen in 14 individuals (14/189; 7.4%) and a switch from CXCR4- to CCR5-use (X4R5) in 3 (3/36; 8.3%). There was no statistical difference between the probability of switching to X4 after a R5 prediction and the probability of switching to R5 after an X4 prediction (p = 0.740). 

### Correlates of CCR5-to-CXCR4 co-receptor switch and clinical impact

**Figure 2:**
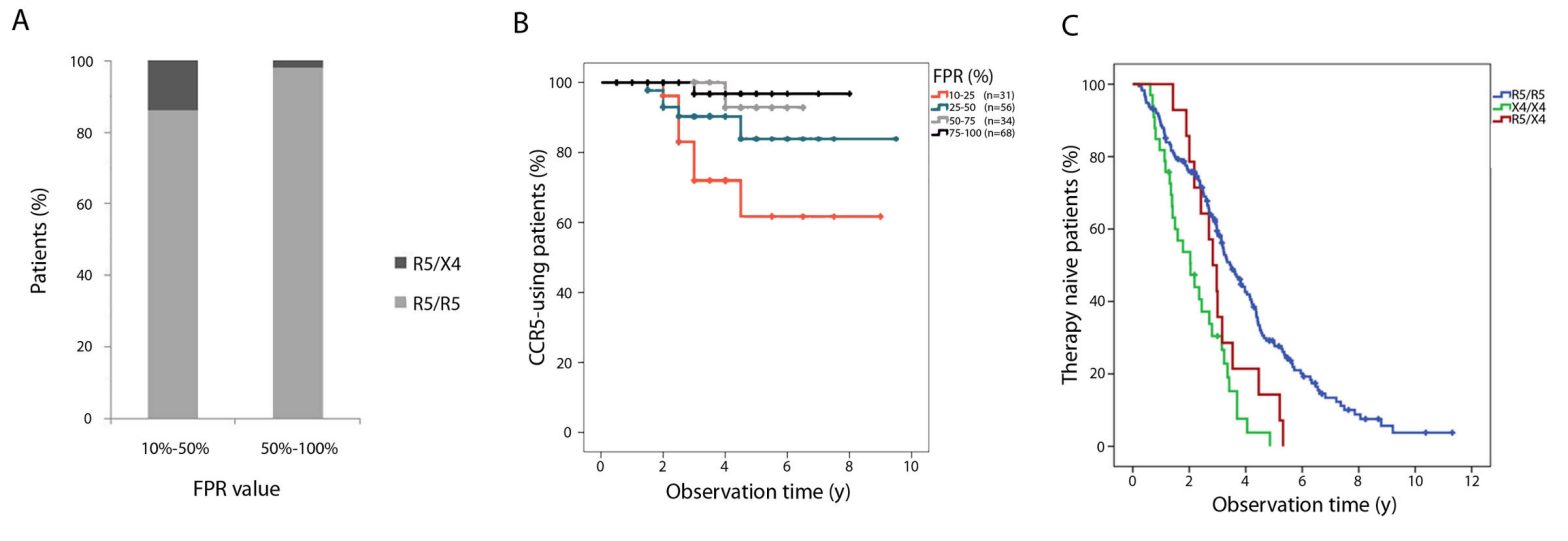
Predictive value of the FPR on co-receptor switch and relation between co-receptor switch and the initiation of ART. Figure **2A**, Percentage of switching (R5X4) and non-switching patients (R5R5) after classification of the patients in two groups according to the FPR at diagnosis (10%-50% and 50%-100%). Figure **2B**, Kaplan-Meier estimates of the percentage of CCR5-use over time. Patients were grouped based on the FPR at diagnosis (10%-25%; 25%-50%; 50%-75%; 75%-100%). Each drop in percentage of CCR5-use reflects a switch to CXCR4-use. n = number of patients in each group. Figure **2C**, Kaplan-Meier estimates of the percentage of therapy naive patients over time for 175 patients with an R5 prediction on both samples (R5R5), 33 patients with an X4 prediction on both samples (X4X4) and 14 patients with a switch from R5 to X4 (R5X4).

The search for clinical predictive markers for co-receptor switch concentrated on the comparison between the 175 R5R5 individuals and the 14 R5X4 individuals. All characteristics and the results of the statistical comparison are shown in Table 1. No difference between the two groups was seen for age, gender, race, CCR5 genotype or transmission route, viral subtype, presence of drug resistance at diagnosis, first viral load or first CD4 count (p > 0.05). Lower FPR at diagnosis was seen in the group with R5X4 switch (median 27.4% versus 59.2% for the R5R5 group; p = 0.001). A significant lower CD4 count at ART initiation - but not at diagnosis - was observed in the R5X4 switchers (p = 0.015). Reanalysis of the data using a cut-off of 5% FPR instead of 10% FPR did not change the associations. Patients with an FPR of less than 50% at diagnosis had a 14% chance to switch co-receptor use over time compared to 2% for patients with an FPR of more than 50% (p = 0.001) (Figure 2A). Of the 14 patients with an R5X4 switch, the FPR at diagnosis was between 10% and 25% in 7 (50%), between 25% and 50% in 5 (35.7%), between 50% and 75% in 1 (7.1%) and between 75% and 100% in 1 (7.1%). 

**Table 1:** Comparison of patient and viral characteristics for the R5R5 control group (n=175) and the R5X4 switchers (n=14).

	**R5** **R5**	**R5** **X4**	
	**n (%)**	**n (%)**	**p-value**
**Patient Characteristics (n = 189)**	175	14	
Age, median (IQR), y (n = 189)	40 (34-46)	41 (31-51)	0.788
Gender, No. (%) (n = 189)	175	14	
Male	144 (82.3%)	11 (78.6%)	0.72
Female	31 (17.7%)	3 (21.4%)	
Race or ethnicity, No. (%) (n = 186)	172	14	
Caucasian	145 (84.3%)	14 (100%)	0.228
Other	27 (15.7%)	0 (0.0%)	
Transmission route, No. (%) (n = 170)	158	12	
Homosexual contact	109 (69.0%)	10 (83.3%)	0.514
Heterosexual contact	43 (27.2%)	1 (8.3%)	0.19
Other	6 (3.8%)	1 (8.3%)	0.407
CCR5 genotype, No. (%) (n = 184)	170	14	
wt/wt	144 (84.7%)	13 (92.9%)	0.697
wt/Δ32	26 (15.3%)	1 (7.1%)	
Therapy initiation, No. (%) (n = 189)	175	14	
Yes	137 (78.3%)	14 (100%)	0.077
No	38 (21.7%)	0 (0.0%)	
CD4^+^ T cell count at diagnosis, Median (IQR), cells/mm^+^ (n = 178)	498 (365-653)	491 (339-590)	0.457
CD4 <350 (n = 40)	36 (22.0%)	4 (28.6%)	0.496
CD4 350 - 500 (n = 50)	47 (28.7%)	3 (21.4%)	0.52
CD4 >500 (n = 88)	81 (49.4%)	7 (50.0%)	0.781
Treatment initiation CD4^+^ T cell count, Median (IQR), cells/mm^+^ (n = 172)	360 (274-482)	227 (159-409)	**0.015**
Drug free period, Mean (IQR), months (n = 152)	32 (15-44)	31 (21-38)	0.947
Follow-up period, Mean (IQR), months (n = 189)	35 (19-46)	31 (21-38)	0.703
**Viral Characteristics**			
Viral load at diagnosis, Median (IQR), log copies/ ml (n = 186)	4.46 (3.95-4.96)	4.43 (3.96-4.77)	0.867
Treatment initiation viral load, Median (IQR), log copies/ ml (n = 183)	4.55 (3.84-4.98)	4.81 (4.24-5.07)	0.297
FPR at diagnosis, Median (IQR), % (n = 189)	59 (31-81)	27 (15-48)	**0.001**
FPR < 50 (n = 85)	73 (41.7%)	12 (85.7%)	**0.001**
FPR > 50 (n = 104)	102 (58.3%)	2 (14.3%)	
Transmitted drug resistance, No. (%) (n = 189)	175	14	
Yes	9 (5.1%)	0 (0%)	1,000
No	166 (94.9%)	14 (100%)	
Virus subtype, No. (%) (n = 189)	175	14	
B	128 (74.9%)	11 (78.6%)	0.411
non B	43 (25.1%)	3 (21.4%)	

For classification as R5 or X4, an FPR cut-off of 10% was applied. For each characteristic, only those patients for whom the information was available were included in the analysis. n: number of included patients; FPR: false positive rate.

The Kaplan-Meier plot in Figure 2B illustrates the rate of tropism switch over time in patients classified according to the FPR at diagnosis. After 1 year of follow-up no R5X4 switches were observed (n = 14), after two years the probability of co-receptor switch was 3.8% in the patients with an FPR between 10% and 25%, 7% in those with an FPR between 25% and 50%, 0% in those with an FPR between 50% and 75% and 0% in those with an FPR between 75% and 100%. After four years the probability of switching was 28%, 9.7%, 7.1% and 3.2%, respectively.

183 of 225 patients (81.3 %) initiated ART during the follow-up period. ART was initiated in all R5X4 switchers (100%) compared to 78.3% of the R5R5 individuals (p = 0.077; Table 1). The Kaplan-Meier plot in Figure 2C illustrates the ART initiation over time. The number of patients initiating therapy within 2 years of diagnosis is the highest in the X4X4 group. After approximately 2 years, an increase in number of patients initiating treatment is observed for the R5X4 individuals, finally resulting in treatment percentages comparable to the X4X4 group. X4X4 patients started ART after a mean of 21 months (IQR: 12-31), compared to 31 months (IQR:21-38) for the R5X4 group and 35 months (IQR:19-46) for the R5R5 group (p < 0.05). Patients with an R5X4 tropism switch had significantly lower CD4^+^ T-cell counts at the time of treatment initiation compared to the R5R5 patients (median 227 cells/mm^3^ versus 360 cells/mm^3^; p = 0.015; Table 1).

### Association between the FPR at diagnosis and the presence of X4 minorities and impact on tropism switch

To evaluate whether a lower FPR at baseline reflects the presence of X4 minorities, DS of V3 was performed. Minority variants were identified in the first sample of 9 of the 14 R5X4 switchers (64.3%) and 2 of the 3 X4R5 switchers (66.6%) (Table 2). 

**Table 2:** Overview of deep sequencing results.

		Population sequencing	Deep sequencing				
	Patient ID	FPR%	Presence of minority X4 variants	# of minority X4 variants	# of reads for each minority variants	% of reads	Outgrowth of minority variants in 2nd sample
R5X4	A	52.5	No	0	0	0	/
	B	89.3	No	0	0	0	/
	C	35.1	Yes	1	11	0.27	N
	E	41.2	Yes	1	27	0.67	N
	F	10.5	Yes	1	11	0.24	N
	G	15	No	0	0	0	/
	H	48	No	0	0	0	/
	I	17	Yes	1	336	6.42	Y
	J	49	No	0	0	0	/
	K	15	Yes	1	10	0.24	/
	L	13.4	Yes	3	303; 55; 52	8.09; 1.47; 1.39	Y
	M	34.6	Yes	3	51; 22; 10	1.37; 0.59; 0.27	N
	O	20.2	Yes	2	12; 11	0.48; 0.44	N
	P	13	Yes	5	17; 10; 9; 7; 7	0.56; 0.33; 0.30; 0.23; 0.23	Y
X4R5	D	6.8	Yes	1	13	0.25	/
	N	3.8	No	0	0	0	/
	Q	7.4	Yes	2	23; 9	0.58; 0.23	Y

For each of the 14 R5X4 and 3 X4R5 switching patients the detection of minority X4 or minority R5 variants respectively is indicated as well as the number of minority variants and the number of reads that this variant represents. # : *number;* % : *percentage.*

The number of unique minority sequences per sample varied between 1 and 5 with a coverage of between 0.23% and 8.09% of the total number of DS reads for the sample (Table 2). A higher prevalence of minor X4 variants was noticed in samples with an FPR of <25% compared to samples with an FPR of >25% (minority X4 variants in 6/7, 85.7% versus 3/7, 42.9%; p = 0.056). No minor variants were detected in the 2 patient with a baseline FPR of >50%. 

**Figure 3:**
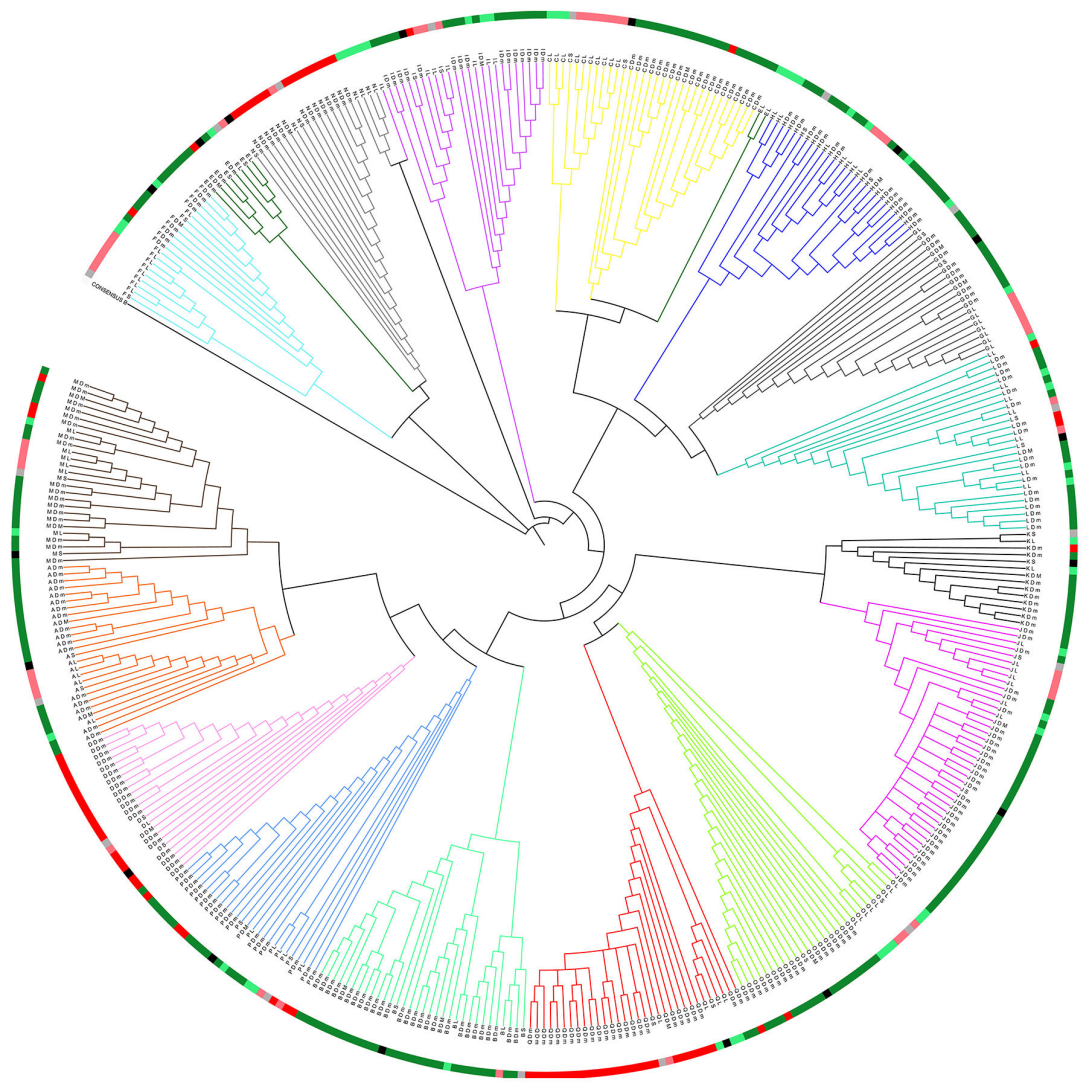
Neighbor-joining tree illustrating the phylogeny of V3 sequences from 17 co-receptor switchers. Sequences were obtained after either population Sanger sequencing on the first (black) and last (grey) plasma sample, deep sequencing on the first plasma sample (dark green: sequence reads predicted as R5; dark red: sequence reads predicted as X4) or single genome sequencing performed on cellular DNA from the last sample ( light green: sequences predicted as R5; light red: sequences predicted as X4). The individual labels contain a patient ID, the sequencing method used to obtain the sequence (S: sanger sequencing; D: deep sequencing; L: limiting dilution sequencing) and, for the deep sequencing reads, an indication of whether it was a major read (M; >10% of all reads) or a minor read (m; <10% of all reads).

For each patient with a tropism switch, all V3 sequences from the DS analysis as well as the V3 sequences obtained after the single genome sequencing and the V3 sequences obtained after population sequencing were aligned for phylogenetic analysis (see Figure 3). The tree topology was suggestive for the outgrowth of a minority X4 strain over time for only 3 of the 14 R5X4 patients (21.4%). For 2 of these 3 patients the minority X4 variant was already present at relatively high frequency in the first sample (6.42% and 8.09%; Table 2). 

### V3 mutational patterns of switching patients

In order to define mutations responsible for tropism shifts, the population V3 sequences of the first and last sample were compared. The number of amino acid replacements varied between 1 and 11. The position of the replacements and the specific substitutions differed between patients but changes at positions 13, 25, 32 and 34 were most frequently observed. The Pearson correlation coefficient for the relation between the FPR and the number of substitutions was calculated but no correlation was found (p = 0.558). Sequences predicted as CXCR4-using had an overall higher net charge than sequences predicted as CCR5-using (mean 6.3 versus 5.0). 

## Discussion

Current guidelines for tropism testing advise clinicians and virologists on how to analyse and interpret the results of co-receptor tropism analyses [1,2]. One question that remains is whether and after what period of time tropism testing should be repeated when there is a considerable time delay between testing and considering the initiation of a CCR5 antagonist.

Overall, the percentage of patients with a tropism switch after a median follow-up of 32 months was low. Of the 175 patients initially predicted as exclusively R5, a switch to X4 was seen in 14 (6.2%). Previous studies have reported between 41% and 50% of CXCR4-use in patients with advanced disease [3,7,12,14,28]. Most of the patients in these studies however were included approximately 8 to 10 years after infection [10,29] while the treatment-free follow-up period in our study was much shorter. 81.3% of the studied patients initiated ART during the study period, 44 (19.8%) in the presence of X4 viruses. If we assume that our patient population reflects the HIV infected population in many Western countries where patients are eligible for antiretroviral drugs as soon as the CD4 count has dropped below 500 cells/mm^3^, the overall percentage of pre-ART CXCR4-use will most probably not exceed 20%.

All co-receptor tropism predictions for this study were performed retrospectively and the outcome was not communicated to the clinicians. The higher number of individuals initiating ART in the R5X4 group and in the X4X4 group compared to patients with R5R5 predictions (100%, 90.9% and 78.3% respectively) can therefore only be explained as a reflection of the worse clinical evolution in these patients. This confirms the generally accepted association between X4 presence and faster disease progression [8,12,14,30]. Whether tropism switch induces the faster immune deterioration or whether X4 viruses can appear because of the decline in immune function remains a topic of debate. The findings of this study support the hypothesis that the co-receptor switch induces the immune decline. Despite identical CD4^+^ T cell counts at diagnosis and comparable follow-up time, patients switching co-receptor use had significantly lower CD4^+^ T cell counts at ART initiation compared to patients that remained R5. Kaplan-Meier plots illustrate the time delay in treatment initiation between the patients diagnosed with X4 virus and patients that switch from R5 to X4, but also reveal the comparable steepness of both curves. 

The observation that 3 of the 36 patients predicted as X4 in the first sample switched to R5 during follow-up is puzzling. Although some have suggested that after infection with a mixed population of R5 and X4 strains X4 viruses might be overgrown by the R5 viruses because they are more prone to CTL responses [31], the support for this hypothesis is weak. It remains questionable whether a true shift in tropism from X4 to R5 can occur and is clinically meaningful. For one X4R5 patient we have reasons to believe that the patient was re-infected with an R5 virus after initial infection with an X4 strain but this remains to be confirmed. Minor R5 sequences were found in the plasma of the first sample collected from the other 2 X4R5 patients but at very low frequency. In only one patient there were indications for a later outgrowth of this initial minor R5 variant. 

In order to identify possible predictive markers for co-receptor tropism switch we compared the baseline characteristics of patients that remained R5 tropic over time and patients that switched from R5 to X4 tropic. No influence of age, gender, race, CCR5 genotype, transmission route, viral subtype, drug resistance, viral load or CD4 count at diagnosis (p > 0.05) was detected, largely confirming previous findings [30,32]. The only parameter that came out as predictive for a subsequent tropism switch was the geno2pheno FPR. Patients with an FPR of <50% are significantly more prone to co-receptor switch than patients with an FPR of >50%, which is in line with previous observations [20]. Little information is available regarding the value of the FPR apart from it being a measure to discriminate R5 and X4 predictions when using the geno2pheno_[co-receptor]_ algorithm. Using DS however, Svicher et al. demonstrated an association between the FPR and the burden of minority X4 variants [33]. Our DS results confirmed this relationship. The importance of minority X4 variants on the later co-receptor tropism switch however remains questionable. Phylogenetic analysis provided evidence for the outgrowth of minority X4 variants over time for 3 of the 14 R5X4 switchers only, including the 2 patients with the highest X4 burden in the first sample. In all other R5X4 switchers the tree topology is more suggestive for evolution from an R5 strain than for the outgrowth of an existing minor X4 variant. 

We next searched for a possible correlation between the number of V3 amino acid substitutions needed for the evolution from R5 to X4 and the FPR but were unable to confirm the hypothesis that a lower FPR reflects a lower genetic barrier for tropism switch. The precise number and or position of amino acid replacements needed for switch remains difficult to define because a high individual variability was observed and tropism specific mutational patterns could not be defined. The number of V3 amino acid differences between the first and last sample in the R5X4 patients varied between 1 and 11, which is in accordance with the results of others describing that at least 2 mutations are needed for a co-receptor tropism switch [34,35]. Positions 13, 25, 32 and 34 were most frequently mutated. The involvement of position 25 in co-receptor prevalence has been recognized long ago but none of the other positions has been reported as implicating co-receptor affinity [36-40]. Predicted CXCR4-using V3 sequences also had an overall higher net positive charge, a specificity also known to be associated with CXCR4-use [36].

Apart from the FPR, all other parameters for which we anticipated a possible influence on co-receptor switch lacked association with early co-receptor switch. Margolis et al. [31] hypothesized that exhaustion of CCR5 positive target cells or shortage in CCR5 receptors could facilitate co-receptor switch. If this hypothesis is true one would expect higher rates of co-receptor switch in patients heterozygous for the CCR5-∆32 deletion because the defective allele results in a reduced concentration of CCR5 molecules on the cell surface [41]. In our patient population however a defective allele was even more frequent in the R5R5 group than in the R5X4 group (15.3% vs. 7.1%).

A limitation of this study is the small number of switching patients that could be included. This may limit the validity of the statistical analysis whereby potential additional correlates of CCR5-to-CXCR4 co-receptor switch could have been missed. Another limitation is that the conclusions are based solely on genotypic analyses. Many have however reported the excellent concordance between phenotypic co-receptor tropism determination and genotypic predictions using geno2pheno [42,43]. Importantly, the conclusion of this work remained even after lowering the FPR cut-off from 10% to 5%. An FPR cut-off of 5% results in a more stringent X4 detection and has been associated with an overall increase in concordance between phenotypic co-receptor tropism determination and genotypic prediction from 85.2% to 91.4% [44]. Also the initial aim of this study was to define the validity in time of a genotypic tropism determination as this is the test most commonly used in clinical practice.

In conclusion, only the FPR result of geno2pheno_[co-receptor]_ tropism prediction was withheld as a significant predictor of a subsequent R5 to X4 switch. The findings of this study show that in patients with R5 prediction at diagnosis and an FPR above 50%, the probability of tropism switch within 2 to 3 years is extremely low. Patients infected with a R5 virus and FPR value below 50% are more prone to co-receptor switch and need repeat testing if initiation of maraviroc is considered and co-receptor tropism was defined more than 1 year before.
